# A Tale of Two Morphs: Modeling Pollen Transfer, Magic Traits, and Reproductive Isolation in Parapatry

**DOI:** 10.1371/journal.pone.0106512

**Published:** 2014-09-11

**Authors:** Benjamin C. Haller, Jurriaan M. de Vos, Barbara Keller, Andrew P. Hendry, Elena Conti

**Affiliations:** 1 Redpath Museum and Deptartment of Biology, McGill University, Montréal, Québec, Canada; 2 Deptartment of Ecology and Evol. Biol., Brown University, Providence, Rhode Island, United States of America; 3 Institute of Systematic Botany, University of Zürich, Zürich, Switzerland; CNRS, France

## Abstract

The evolution of the flower is commonly thought to have spurred angiosperm diversification. Similarly, particular floral traits might have promoted diversification within specific angiosperm clades. We hypothesize that traits promoting the precise positional transfer of pollen between flowers might promote diversification. In particular, precise pollen transfer might produce partial reproductive isolation that facilitates adaptive divergence between parapatric populations differing in their reproductive-organ positions. We investigate this hypothesis with an individual-based model of pollen transfer dynamics associated with heterostyly, a floral syndrome that depends on precise pollen transfer. Our model shows that precise pollen transfer can cause sexual selection leading to divergence in reproductive-organ positions between populations served by different pollinators, pleiotropically causing an increase in reproductive isolation through a “magic trait” mechanism. Furthermore, this increased reproductive isolation facilitates adaptive divergence between the populations in an unlinked, ecologically selected trait. In a different pollination scenario, however, precise pollen transfer causes a decrease in adaptive divergence by promoting asymmetric gene flow. Our results highlight the idea that magic traits are not “magic” in isolation; in particular, the effect size of magic traits in speciation depends on the external environment, and also on other traits that modify the strength of the magic trait's influence on non-random mating. Overall, we show that the evolutionary consequences of pollen transfer dynamics can depend strongly on the available pollinator fauna and on the morphological fit between flowers and pollinators. Furthermore, our results illustrate the potential importance of even weak reproductive isolating barriers in facilitating adaptive divergence.

## Introduction

Adaptive radiations are often attributed to particular traits that promote divergence into under-utilized ecological niches [1]–[3]. For example, the development of the flower might have spurred angiosperm diversification through plant–pollinator interactions that afforded new possibilities for reproductive isolation and adaptive differentiation [2], [4]–[9]. Moreover, particular floral traits, such as nectar spurs, bilateral symmetry (zygomorphy), and scent, might have promoted diversification of particular clades by providing further mechanisms for reproductive isolation [10]–[13]. Traits that influence pollinator choice among flowers, such as scent and color, might produce behavioral isolation, whereas traits that affect the morphology of the flower and its interaction with the pollinator's body, such as zygomorphy, might produce mechanical isolation [14], [15].

In one type of mechanical isolation, termed the “*Pedicularis* type” by Grant [14], reproductive isolation depends on the precision with which pollen is transferred via different, specific positions on the bodies of pollinators [e.g., 16], rendering flowers with different sexual organ positions reproductively isolated from each other. Effects of such “precise pollen transfer” [17]–[20] on reproductive isolation have been explored mainly in the context of zygomorphy, a floral trait believed to increase the precision of pollen transfer [10], [13], [19], [21], [22]. Brantjes [23], for example, found that placement of pollen at sites only 2 mm apart on the pollinator produced complete reproductive isolation between sympatric *Polygala* species. Heterostyly, a floral syndrome characterized by flowers that differ in the reciprocal placement of male and female sexual organs, is also thought to promote precise pollen transfer [24]–[27], but possible effects of heterostyly on reproductive isolation have received little attention [28].

Because floral traits that are thought to promote precise pollen transfer might thus facilitate partial or complete reproductive isolation among closely related species [14], [29]–[33], such traits might contribute to driving diversification. In support of this prediction, phylogenetic tests in different angiosperm clades have linked the evolution of zygomorphy and heterostyly to increased diversification rates [10], [19], [34], [35]. However, such macro-evolutionary analyses cannot disentangle the role of precise pollen transfer from effects due to pollinator specialization and increased outcrossing, which might also influence diversification [19], [35]. Furthermore, experimental studies of how the dynamics and precision of pollen transfer might affect reproductive isolation and diversification are lacking, because tracking and manipulating pollen movement in natural systems [e.g., 36] is challenging due to the necessity of manually counting thousands of pollen grains for a sufficient number of pollen-transfer events and inter-specific comparisons [37], [38]. Given these difficulties with phylogenetic and experimental approaches, our study employs modeling to explore how the dynamics and precision of pollen transfer affect reproductive isolation and adaptive divergence.

**Figure 1:**
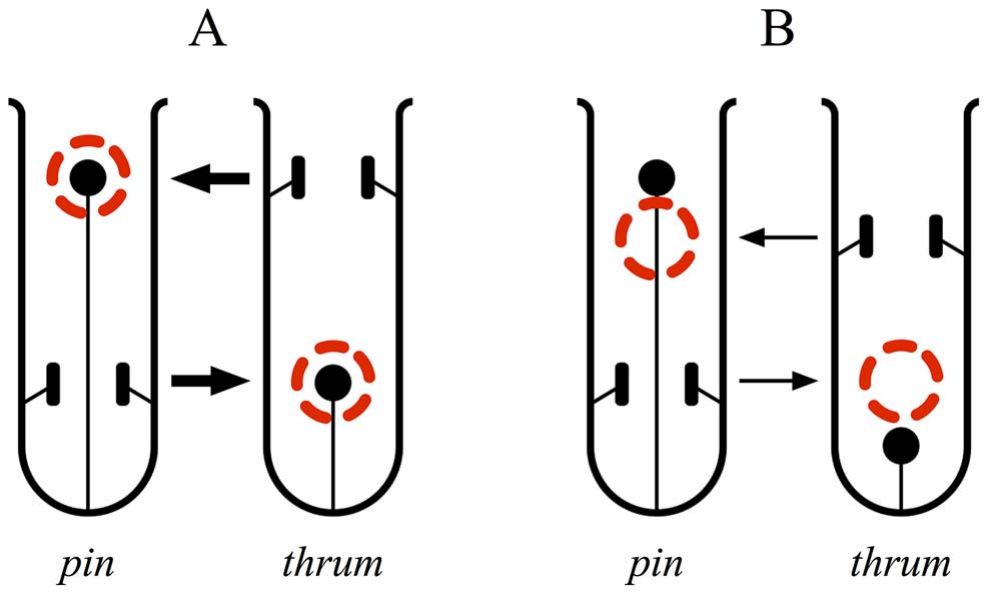
Conceptual “cartoons” of the effects of reproductive-organ height on the transfer of pollen between distylous flowers. Arrows show directions of pollen flow, arrow widths show magnitude of expected fertilization, and dashed red circles indicate the region with the highest probability of pollen deposition. A: Pollen transfer between well-matched reciprocal morphs. Pollen donated at the low position by a pin is transferred to a low position on the pollinator's body and arrives at a low position in the recipient thrum; similarly, pollen donated at a high position by a thrum arrives at a high position in the recipient pin. Because pollen arrives at the height of the recipient stigma and is compatible with it, fertilization is likely to occur. B: Hindrance of pollen transfer between reciprocal morphs poorly matched in their reproductive-organ heights. Due to this mismatch, pollen arrives at the wrong height and is thus less likely to be received by the stigma and result in fertilization. The height mismatch thus causes some degree of reproductive isolation.

Heterostyly, a floral syndrome [reviewed by 26,27,39,40] characterized by a morphological component (reciprocal herkogamy) and typically also a physiological component (sporophytic self- and intra-morph incompatibility), is well-suited to model the role of precise pollen transfer in mechanical isolation and speciation. Reciprocal herkogamy is the reciprocal positioning of anthers and stigmas in two (distyly) or three (tristyly) floral morphs. We will here focus on distyly, in which “pins” (“L-morphs”) have a high stigma and low anthers, whereas “thrums” (“S-morphs”) have a low stigma and high anthers. Reciprocal herkogamy promotes inter-morph pollen transfer and reduces intra-morph and intra-flower transfer [37]. These effects depend on (somewhat) precise transfer of pollen: pollen picked up at a given corolla-tube height tends to be deposited at a similar height in the next flower visited [27]. More specifically, pollen picked up from a pin (thus at the low position) will tend to be delivered to a low-positioned stigma (that of a thrum), where it is compatible, rather than to another pin's stigma, where it would be incompatible; and the same is true, correspondingly, for transfer from thrum to pin at the high position (Fig. 1A). Outcrossing is thus promoted by reciprocal herkogamy, and is also enforced by the diallelic self-incompatibility system of distyly [17]. Reciprocal herkogamy also reduces sexual interference, i.e., conflict between the male and female functions of the flower [reviewed in 17], by reducing wastage of pollen on incompatible stigmas and “clogging” of stigmas with incompatible pollen [37], [41]–[48].

Keller et al. [28] proposed that the combination of reciprocal herkogamy and precise pollen transfer could contribute to reproductive isolation between populations or species with different sexual organ positions [or different corolla lengths; 49]. In particular, the degree of spatial matching between the positions of reciprocal reproductive organs might affect the likelihood of pollen transfer between flowers, because two well-matched flowers might exchange pollen more effectively than two poorly matched flowers (Fig. 1). Therefore, even small differences in reproductive-organ heights might contribute to reproductive isolation. Furthermore, this effect on non-random mating means that divergent selection on these height traits, exerted by different local pollinators, might cause the traits to act as “magic traits” strongly promoting speciation [50], [51].

Although the precision of pollen transfer might be insufficient to produce complete reproductive isolation [52], partial precision in transfer might suffice to produce partial isolation [49]. Even relatively minor barriers to gene flow might contribute to adaptive divergence and speciation – particularly when combined with other barriers, when acting early in the process of reproduction (as mechanical isolating barriers do), and when arising early in the process of divergence [53]–[56]. Precise pollen transfer might thus facilitate greater adaptive divergence between populations in different environments [53], [57], which we set out to test in this study.

### Our study

There is a long history to the idea that plant–pollinator interactions have driven angiosperm diversification, but surprisingly few studies have explored the details of this idea, and mechanical isolation has been particularly neglected. We here test the hypothesis that precise pollen transfer can produce mechanical reproductive isolation between populations differing in their reproductive-organ positions, and that this reproductive isolation can enable greater adaptive divergence in whatever other traits might be subject to divergent ecological selection. We test this hypothesis with a mechanistically detailed model of the evolution of heterostylous plants occupying two parapatric patches that are ecologically different (thus supplying the divergent ecological selection necessary to test our hypothesis). Our model uses an individual-based approach to explicitly simulate pollen flow dynamics, accounting for the effects of sexual interference, reproductive-organ positioning, and pollen transfer precision and stochasticity. Possible variation in pollinator morphology that might influence pollen flow is also modeled, using “pollinator functions” that represent the stickiness of a pollinator's body at different positions.

We chose to model heterostyly because it minimizes confounding effects due to sexual interference [17], and because heterostyly offers rich opportunities to model little-explored details of plant–pollinator interactions, including the possibility of morph-specific effects of particular pollinator morphologies. There is also empirical evidence for precise pollen transfer and differential positioning of reproductive organs between heterostylous species [17], [28], [37], [47], [58], and even some knowledge of the underlying genetics to guide our model design [27], [59]–[61].

## Methods

### Model overview

A full description of the model is given online (Appendix S1), and we here present a brief overview. Model parameters are shown in Table 1, while individual-level traits of the modeled plants are shown in Table 2.

**Table 1:** Model parameters with their symbols and values.

	Symbol	Value
Population carrying capacity	*K*	1000
Initial value for the three genetic traits	*x*_i_, *y*_i_, *z*_i_	0.5
Initial genetic variance for the reproductive-organ–height traits	σ_xi_^2^, σ_yi_^2^	0.006
Probability of a mutation occurring	*µ*	0.1
Standard deviation of the mutation effect size	*α*	0.1
Number of ovules per flower	*n*_o_	50
Number of pollen grains per flower	*n*_p_	1000
Number of received pollen grains that results in complete style clogging	*n*_s_	250
Uptake probability for each pollen grain in pollination (transfer between flowers)	*u*_p_	0.1
Uptake probability for each pollen grain in self-transfer	*u*_s_	0.1
Mortality probability per year	*m*	0.25
Ecological trait optimum for environment 1	*θ*_1_	0.0
Ecological trait optimum for environment 2	*θ*_2_	1.0
Season length (pollination events per year)	*v*	10000
Standard deviation of pollen height stochasticity during pollen transfer between flowers	*σ*_j_	0.01, 0.1, 0.5
Pollinator crossover probability	*c*	0.0, 0.001, 0.01, 0.05, 0.1, 0.2, 0.35, 0.5
Strength of ecological selection (standard deviation of the fitness function)	*ω*	0.25, 0.5, 1, 5
Pollinator functions for patch 1 and 2, giving the probability that pollen will stick to a pollinator at height *h*	*π*_1_(*h*), *π*_2_(*h*)	(control^a^), (uniform, high-biased), (bimodal 1, bimodal 2)
Gaussian pollen transfer gap	*σ*_p_	0.1^b^
Gaussian pollen self-transfer gap	*σ*_s_	0.1^b^
Lognormal pollen transfer gap	*l*_g_	0.2^c^
Lognormal pollen transfer function shape parameter	*l*_σ_	1.0^c^

a Control realizations did not use the pollinator functions, and involved completely imprecise pollen transfer; see Methods, *Model summary*, and Appendix S1, *Pollination phase*, for details.

b Used only for the Gaussian pollen transfer version of the model; see Appendix S1, *Pollination phase*.

c Used only for the lognormal pollen transfer version of the model; see Appendix S1, *Pollination phase*.

**Table 2:** Individual traits with their symbols and permissible values.

	Symbol	Value
Reproductive-organ position 1; a stigma exists at this height if S = 0, or anthers if S = 1	*x*	[0.0, 1.0]
Reproductive-organ position 2; a stigma exists at this height if S = 1, or anthers if S = 0	*y*	[0.0, 1.0]
Ecological trait, influencing adaptation to the local patch's ecological optimum (θ_1_ or θ_2_)	*z*	any
Morph-determining trait, governing reproductive-organ development and also the legitimacy of crosses	*S*	(0, 1)
Number of remaining unfertilized ovules	*o*	0–*n*_o_
Number of remaining pollen grains	*p*	0–*n*_p_
Style clogging index, indicating the degree to which the style has become clogged by pollen tubes	*s*	0–*n*_s_

Values are listed as an interval [a, b], a set of discrete values (a, b), a range of integer values a–b, or “any” to indicate that all real values are allowed. Traits above the separating line (*x*, *y*, *z*, *S*) are genetic (heritable, and immutable for any given individual); traits below the line (*o*, *p*, *s*) are non-genetic (not heritable, and subject to change for each individual over time).

The model is an individual-based evolutionary simulation of distylous flowers of perennial plants in two parapatric patches. Each year in the model comprises germination, mortality, and pollination phases, described below. The two patches have no internal spatial structure, but they differ ecologically in an unspecified way, producing stabilizing natural selection toward a different optimum value in each patch (*θ*_1_ vs. *θ*_2_) for a quantitative genetic “ecological trait” of the plants, *z*. Adaptive divergence is opposed by gene flow due to “pollinator crossover” events in which pollinators pick up pollen from a flower in one patch and deliver it to a flower in the other. The extent of gene flow depends on the pollinator crossover probability, *c*, which models levels of geographic isolation ranging from allopatry (*c* = 0.0) to sympatry (*c* = 0.5). Gene flow also depends on the mechanistic details of pollen transfer, which is modeled at the level of the movement of individual pollen grains (see below).

The dynamics of pollen transfer affect reproductive success: plants that deliver or receive fewer compatible pollen grains will produce fewer offspring. The pollen transfer dynamics depend on floral morphology, and therefore the modeled floral morphological traits (described below) are subject to sexual selection mediated by the pollinators [62]. This situation for floral traits is in contrast to the “ecological trait” mentioned above, which is subject to natural selection unrelated to pollination. The model thus incorporates both ecological and sexual selection (on separate traits), and investigates how they jointly influence gene flow to determine the degree of adaptive divergence in the naturally selected “ecological trait”.

For simplicity, the plants are modeled as having a single flower (see *Conclusions* for a discussion of model assumptions). Quantitative genetic traits, *x* and *y*, govern the particular heights at which the anthers and stigma are located within the corolla tube of the flower. The plants also possess an unlinked diallelic trait *S* with Mendelian inheritance, similar to the S-locus of heterostylous plants [27], that governs both complete heteromorphic incompatibility and the “polarity” of the traits controlling reproductive-organ heights (whether *x* determines anther height and *y* determines stigma height, or vice versa). One *S* allele thus represents “pins” and the other represents “thrums” (Fig. 1), but the sense of this polarity – which *S* allele represents which morph – is emergent rather than specified in the model's design. These genetic details are in agreement with current knowledge of heterostyly; see Appendix S1, *Environment and state variables*, for further discussion.

**Figure 2:**
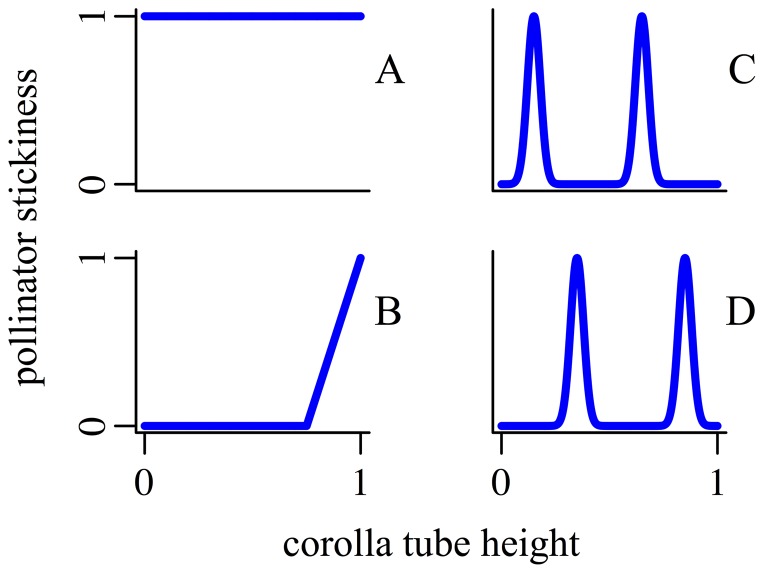
Pollinator stickiness functions used in the presented results. The *x*-axis represents the corolla tube height (0 = bottom, 1 = top) at which the pollen grain encounters the pollinator's body. The *y*-axis represents the probability that the pollen grain will stick to the pollinator at that height. A: The “uniform” pollinator, with equal, maximal stickiness at all heights. B: The “high-biased” pollinator, which is not sticky at all below a threshold height, and then is increasingly sticky with increasing height. C: The “bimodal-low” pollinator, which is sticky principally at two distinct positions on its body. D: The “bimodal-high” pollinator, which is sticky principally at two distinct positions different from those of the “bimodal-low” pollinator.

Pollen transfer dynamics also depend on pollinator morphology. Each patch has a native pollinator representing a morphologically homogeneous pollinator fauna, which can be interpreted as the “most effective pollinator” for the patch in a mixed-pollinator milieu [63]. Pollinators are represented by “pollinator functions”, denoted *π*_1_(*h*) and *π*_2_(*h*) for patches 1 and 2 respectively. The pollinator function determines the probability that pollen will stick to a pollinator's body (or its proboscis, its tongue, etc.) at a given corolla-tube height *h*. Pollinators in the model are otherwise unspecified and are unaffected by model dynamics; in particular, the pollinators do not evolve because no selective pressures involving attraction or reward exist in the model. The pollinator functions used are shown in Fig. 2, and include a “uniform” pollinator that is equally sticky everywhere, a “high-biased” pollinator that is sticky only at positions that contact the flower near the top of the corolla tube, and two different “bimodal” pollinators, each sticky at two particular positions. Although little is known of the effects of pollinator morphology on pollen transfer dynamics, these alternative functions were developed to represent biologically realistic possibilities (see Appendix S1, *Parameters*).

Pollination is the last phase in each year, but will be described first here. Each year, every plant has a limited number of ovules, *o*, that can be fertilized, and a limited number of pollen grains, *p*, that it can donate (*n*_o_ and *n*_p_, respectively, at the beginning of the year). The pollination phase is broken into *v* separate pollination events, each consisting of several steps. In the first step, the pollinator visits a randomly chosen donor flower and removes pollen grains, each grain with a probability *u*_p_. During removal, the height of each pollen grain is perturbed stochastically (e.g., through pollinator movements), with a standard deviation of perturbation *σ*_j_ that represents the precision of pollen transfer. Each pollen grain then sticks to the pollinator's body with a probability given by the pollinator function evaluated at the pollen grain's perturbed height. Pollen that sticks is transported to a randomly chosen recipient flower (which might be in the other patch, if pollinator “crossover” occurs between patches), where it is perturbed in height using *σ*_j_ as before to produce a final height (the net effect of *σ*_j_ on pollen grain height is shown in Fig. S2). The pollen grain is then delivered to the recipient's corolla tube at that final height. Whether pollen is received by the recipient's stigma depends on the difference between the pollen grain's final height and the stigma height, relative to a scaling factor *σ*_p_; pollen delivered close to the stigma is more likely to be received. Finally, pollen received by the stigma might cause fertilization (if it is compatible), with a probability inversely proportional to the extent of “style clogging”, *s*, in the recipient flower. When *n*_s_ pollen grains have been received by a flower, its style is fully clogged and fertilization is completely blocked.

Pollination events can also result in the transfer of self pollen from anthers to stigma, conceptually as a result of the pollinator jostling the flower. This self-transfer cannot result in fertilization (due to pollen incompatibility), but it does cause wastage of pollen and style clogging. For each pollen grain in the flower, the probability of self-transfer depends on the height differential between anthers and stigma in the flower (the probability of self-transfer decreases with increasing anther-stigma separation, relative to a scaling factor *σ*_s_), with a base self-transfer probability of *u*_s_ with no anther-stigma separation.

Fertilized ovules develop into seedlings during the germination phase of the following year, with trait values based on sexual reproduction of the parents, modified by mutation occurring at rate *µ* with an effect size standard deviation of *α*. Although many seedlings can be produced, the adult population of each patch is limited to a carrying capacity *K*; typically most seedlings do not survive the germination phase to become adults. The probability of maturation to adulthood depends on the ecological fitness of each seedling, as determined by the difference between the seedling's *z*, the patch optimum *θ*, and the strength of ecological selection *ω* (the width of the stabilizing fitness function). See Appendix S1, *Germination phase*, for further details.

Seedlings die due to ecological selection during the germination phase, as described above. During the mortality phase, on the other hand, adult plants experience random mortality with probability *m*, representing deaths due to old age, herbivory, and bad luck. This mortality generates space that will be filled by seedlings in the next year's germination phase.

Each model realization begins with each population having a unimodal distribution of *x* and *y* values centered at the middle of the corolla tube, and with an equal probability for the two *S* alleles. This represents a state in which the genetic framework for heterostyly exists, but differentiation into a well-defined dimorphism of pins and thrums as determined by *S* has not yet occurred. This state is not intended to be biologically meaningful, since our model is not intended to capture the emergence of heterostyly from an ancestral non-heterostylous state [e.g., 64,65,66]; it is merely an unbiased initial state from which dimorphic heterostyly can emerge. Each plant begins with a value for the ecological trait *z* that is midway between the two patch optima, and is thus equally maladapted to both patches.

Finally, reference will be made to “control” realizations of the model. The control realizations establish the expected outcome without precise pollen transfer, as a baseline for comparison to the effects of precise pollen transfer in the “treatment” realizations. In the control realizations, the pollinator functions, stochastic pollen height deviations, and use of the pollen delivery height and stigma height in calculating the probability of pollen delivery are all disabled (see Appendix S1, *Pollination phase*, for further details). The net effect for these realizations is that the probability of pollen delivery from donor anthers to recipient stigmas does not depend on their respective positions or on pollinator morphology.

### Model execution

Five parameters were varied in model realizations: the precision of pollen transfer *σ*_j_, the pollinator crossover probability *c*, the strength of ecological selection *ω*, and the pollinator functions *π*_1_ and *π*_2_ (Table 1). For each combination of parameters, 150 realizations were conducted, for a total of 43200 realizations. For each realization, the model was executed for 10000 generations, which was sufficient for it to equilibrate within the range of stochastic transient dynamics. Dimorphism in reproductive-organ heights evolved quickly from the initial unimodal state (∼100 generations; Fig. S1), because reciprocal herkogamy decreased sexual interference due to self- and intra-morph pollination (see Introduction).

From the infinitude of possible pollinator functions for the two patches, we chose three biologically relevant pollination scenarios to investigate (Table 1). In scenario 1, one patch used the “bimodal-low” pollinator (Fig. 2C), whereas the other used the “bimodal-high” pollinator (Fig. 2D). These pollinators produced pollinator-mediated sexual selection for divergent reproductive-organ positions, allowing us to explore the role of “magic” reproduce-organ–position traits in driving adaptive divergence. In scenario 2, one patch used the “uniform” pollinator (Fig. 2A) and the other used the “high-biased” pollinator (Fig. 2B). These pollinators produced a difference between the patches in pollinator service at the low reproductive-organ position, allowing us to look at the evolutionary consequences of morph-specific effects of pollinator morphology (e.g., Beach & Bawa, 1980). In scenario 3, both patches used the “uniform” pollinator, removing pollinator-mediated divergent sexual selection. This allowed us to test for a “reinforcement-like” effect of reproductive character displacement in response to strong divergent natural selection [67]. We found no evidence of such an effect, and so results of scenario 3 are not reported. The motivation behind the other two scenarios is considered in greater detail in the Discussion, since our results help to illuminate the purpose of these scenarios. Evidence for the biological relevance of the pollinator functions used is presented in Appendix S1, *Parameters*.

### Data analysis

The final state of the realizations was evaluated with three metrics. “Ecological divergence”, 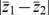, represents the magnitude of divergent local adaptation in the naturally selected “ecological trait” *z* to the patch optima *θ*_1_ and *θ*_2_. “Isolation at fertilization” measures aggregate reproductive isolation due to both geographic separation of the patches and sexual selection against non-resident pollen, calculated as the number of ovules fertilized by resident pollen divided by the total number of ovules fertilized. Finally, “organ mismatch”, 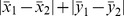, represents the magnitude of divergence between patches in the mean heights of corresponding reproductive organs [following 68]. Analysis based on these metrics (and a few others described where presented) was conducted using R [version 2.15.1; 69]. A dataset containing results from all model realizations is available on Dryad (REF).

## Results

### Scenario 1: Different bimodal pollinators

**Figure 3:**
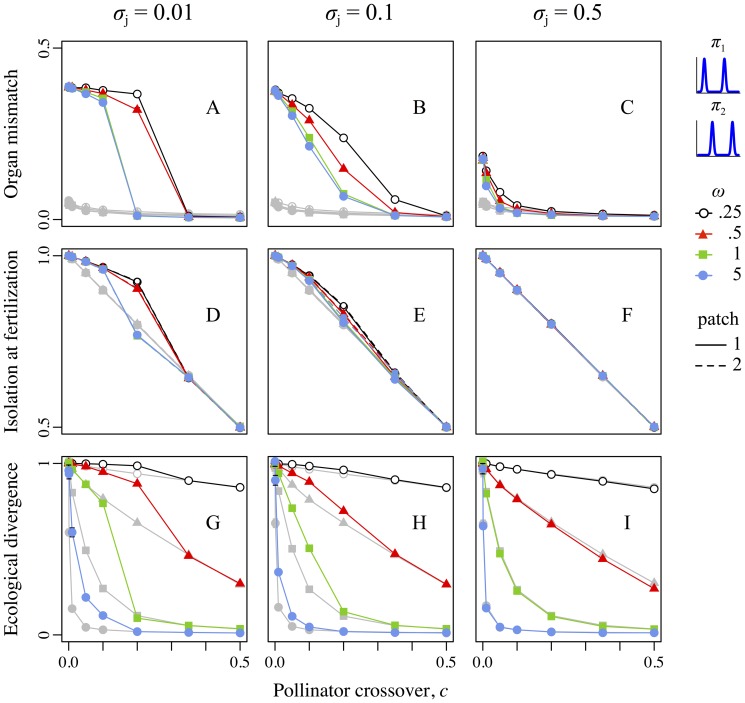
Reproductive-organ–height mismatch, reproductive isolation at fertilization, and ecological divergence as a function of the pollinator crossover probability, strength of selection, and precision of pollen transfer for scenario 1, involving the pollinator pair “bimodal-low” + “bimodal-high”. For all panels, colors and plot symbols represent the strength of selection. For panels D–F, line dashing indicates the patch depicted (1 or 2), but the two patches respond essentially identically in this scenario. Columns correspond to levels of pollen transfer precision: left is precise, *σ*_j_ = 0.01; center is intermediate, *σ*_j_ = 0.1; right is imprecise, *σ*_j_ = 0.5. The *x*-axis in all panels represents the pollinator crossover probability, *c*, from allopatry (*c* = 0.0) to sympatry (*c* = 0.5). Gray lines and symbols in all panels show the control runs corresponding to the (colored) treatment runs. Error bars show ±*SE*, which is often too small to be visible. Top row (A–C): The *y*-axis shows the magnitude of spatial mismatch between reciprocally placed sexual organs of the two floral morphs. Center row (D–F): The *y*-axis shows the degree of reproductive isolation present at fertilization, an indication of the strength of sexual selection against non-local pollen (i.e., mechanical isolation); note this metric also includes the temporally prior effect of geographic isolation. Bottom row (G–I): The *y*-axis shows the extent of ecological divergence between populations.

The difference in pollinators here often produced divergence in reproductive-organ positions between the patches (Figs. 3A–C). This positional mismatch contributed to mechanical reproductive isolation (Figs. 3D–F), which allowed enhanced divergence in the ecological trait *z*, producing greater adaptive divergence in treatment realizations compared to controls (Figs. 3G–I). The increase in adaptive divergence was greatest for intermediate pollinator crossover probabilities (0.001<*c*<0.2), because very high crossover precluded divergence in reproductive-organ positions (Figs. 3A–C) whereas very low crossover allowed adaptive divergence even in the control realizations due to geographic isolation alone (Figs. 3D–I). The increase in divergence was greatest for weaker ecological selection (particularly *ω*≥1), because strong ecological selection produced high divergence regardless of gene flow (Figs. 3G–I). Finally, the increase in divergence was greatest with very precise pollen transfer (*σ*_j_ = 0.01) and absent with very imprecise transfer (*σ*_j_ = 0.5), because precise pollen transfer maximized the effect of divergent reproductive-organ positions on reproductive isolation (Figs. 3D–F). When all three factors aligned (intermediate crossover probability, weak ecological selection, and very precise pollen transfer), adaptive divergence in treatment realizations could be several times greater than in the corresponding control realizations. In absolute terms, treatment realizations sometimes increased divergence over corresponding controls by more than half of the total difference between the patch optima (Fig. 3G).

### Scenario 2: The uniform and high-biased pollinators

**Figure 4:**
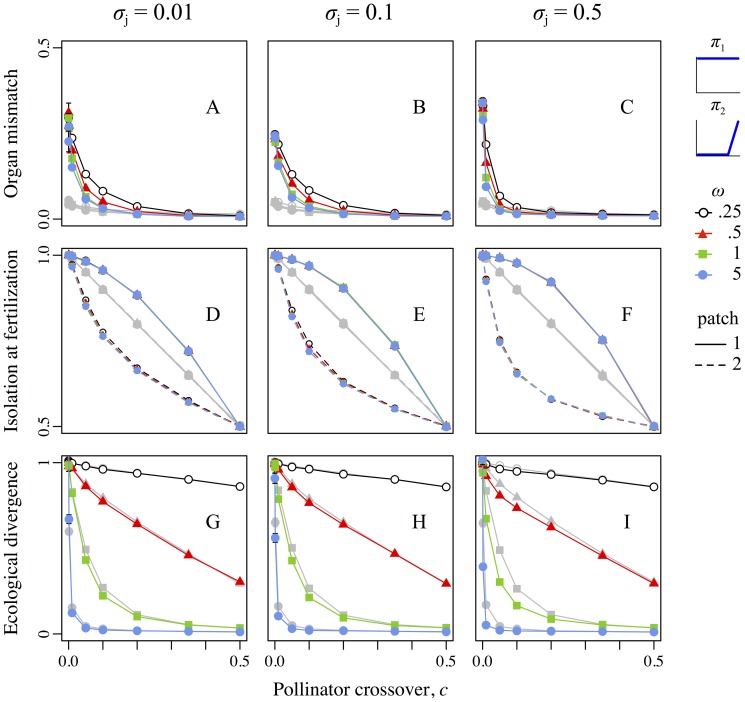
Reproductive-organ–height mismatch, reproductive isolation at fertilization, and ecological divergence as a function of the pollinator crossover probability, strength of selection, and precision of pollen transfer for scenario 2, involving the pollinator pair “uniform” + “high-biased”. Colors, symbols, error bars, dashing, columns, rows, and axes are as in Fig. 3.

As in scenario 1, divergence in reproductive-organ positions here occurred for low pollinator crossover probabilities (Figs. 4A–C). Unlike in scenario 1, however, mechanical reproductive isolation differed between patches: compared to controls, isolation was higher in the “uniform”-pollinator patch, but lower in the “high-biased”–pollinator patch, and this was the case for all levels of pollen transfer precision (Figs. 4D–F). Overall, ecological divergence in this scenario was lower for treatment realizations than for controls (Figs. 4G–I), despite the divergence in reproductive-organ positions. This maladaptation was greatest at intermediate pollinator crossover probabilities (0.001<*c*<0.2), because high crossover meant that the two populations essentially shared a single pollinator milieu (no pollinator-specific effects), whereas low crossover allowed adaptive divergence even in the control realizations due to geographic isolation alone (Figs. 4D–F). Maladaptation was greater with weaker ecological selection (*ω*≥1), because strong ecological selection produced high divergence regardless of gene flow (Figs. 4G–I). Finally, maladaptation was strongest with very imprecise pollen transfer (*σ*_j_ = 0.5), because this magnified pollinator-specific effects (see Discussion, *Scenario 2*). When all three factors aligned (intermediate crossover rate, weak ecological selection, and very imprecise pollen transfer), adaptive divergence in treatment realizations sometimes decreased by more than half relative to the corresponding controls. In absolute terms, a decrease in adaptive divergence of up to roughly a quarter of the total difference between the patch optima was observed (Fig. 4I).

**Figure 5:**
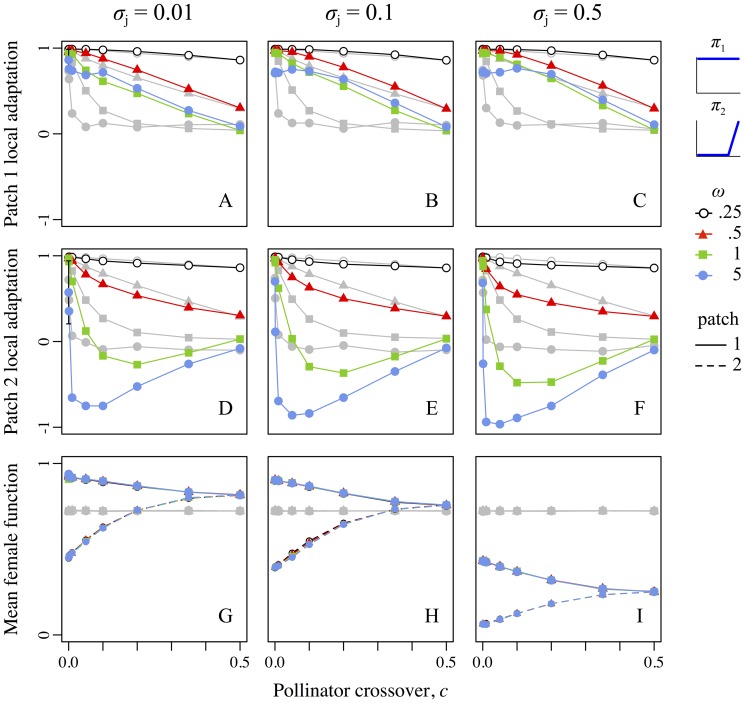
Asymmetrical dynamics of adaptation and reproductive function for scenario 2, involving the pollinator pair “uniform” + “high-biased”. Colors, symbols, error bars, dashing, and columns are as in Fig. 3. The *x*-axis in all panels represents the pollinator crossover probability, *c*, from allopatry (*c* = 0.0) to sympatry (*c* = 0.5). Top row (A–C): The *y*-axis shows the degree of adaptation to the local optimum in patch 1, 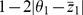, which ranges from complete local adaptation (+1.0) to complete maladaptation (−1.0; e.g., complete adaptation to the optimum of the other patch). Middle row (D–F): The *y*-axis shows the degree of adaptation to the local optimum in patch 2, 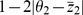, ranging from +1.0 to−1.0 as for the top row. Bottom row (G–I): The *y*-axis shows the mean female function, calculated as the percentage of available ovules filled at the end of the pollination phase.

To better explain the dynamics of this scenario, we use Fig. 5 to present some additional results: patch-specific local adaptation and mean female function. Adaptation to the local optimum in the “uniform”-pollinator patch, 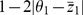, was usually higher for treatment realizations than for controls (Figs. 5A–C). However, the adaptation in the “high-biased”–pollinator patch, 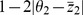, was usually much lower for treatment realizations than for controls (Figs. 5D–F). The net effect of somewhat increased adaptation in one patch, but greatly decreased adaptation in the other, is the net maladaptation described above and shown in Figs. 4G–I.

Finally, mean female function (fraction of ovules fertilized) was divergent between patches, with the “uniform”-pollinator patch generally experiencing a higher fertilization rate than the “high-biased”–pollinator patch (Figs. 5G–I). The extent of divergence in fertilization increased with decreasing pollinator crossover. A marked decrease in female function was observed in treatment realizations with very imprecise pollen transfer, *σ*_j_ = 0.5 (Fig. 5I), which was a consequence of pollen limitation due to the highly stochastic pollen transfer dynamics. Note, however, that differences in female function did not substantially influence the above results, because fertilization was sufficient to maintain population size (results not shown).

### Other sources of reproductive isolation

**Figure 6:**
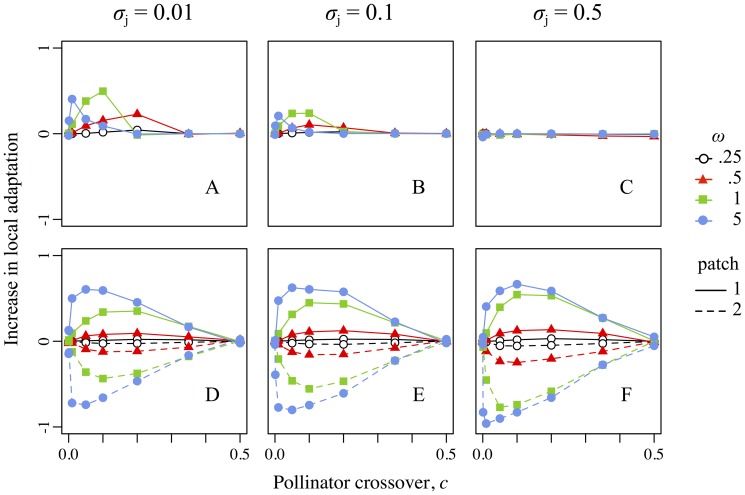
Increase in local adaptation as a function of the pollinator crossover probability, strength of selection, and precision of pollen transfer for both scenarios. Top row (A–C): scenario 1, involving the pollinator pair “bimodal-low” + “bimodal-high”. Bottom row (D–F): scenario 2, involving the pollinator pair “uniform” + “high-biased”. The increase in local adaptation is defined as the difference between the mean local adaptation in treatment realizations and the mean local adaptation in corresponding control realizations with the same parameter values (where the local adaptation in patch *i* is defined as 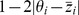, as in Fig. 5). Positive and negative values thus represent increased and decreased adaptation, respectively, in treatment realizations relative to controls. Colors, symbols, error bars, dashing, columns, rows, and axes are as in Fig. 3.

Figs. 3D–F and 4D–F showed reproductive isolation at fertilization, combining the effects of geographic isolation (due to the pollinator crossover probability) and mechanical isolation (due to mismatched reproductive-organ positions and precise pollen transfer). Other factors, such as ecological and sexual selection against hybrids and backcrosses, can also influence reproductive isolation and subsequent adaptive divergence. These effects are difficult to quantify, because “resident” versus “hybrid” is not clearly defined when every individual likely has backcrossed ancestry. Nevertheless, the net effect of all such factors – the total effect size of heterostyly and precise pollen transfer on adaptive divergence – is shown by the relative increase (or decrease) in local adaptation in treatment realizations compared to control realizations (Fig. 6).

## Discussion

We used an individual-based model to explore the hypothesis that floral traits that promote precise pollen transfer could spur diversification by allowing mechanical reproductive isolation, and consequently adaptive divergence, to emerge between populations differing in their reproductive-organ positions [14], [28], [32]. As hypothesized, precise pollen transfer contributed – with certain pollinators and under certain conditions – to reproductive isolation and thus facilitated adaptive divergence relative to control realizations without precise pollen transfer. This outcome strongly depended on the pollinator fauna, however, because the opposite effect (a decrease in adaptive divergence relative to controls) was observed with a different pair of pollinators.

### Scenario 1: Magic traits, magic modifiers, and magic environments

A scenario with “bimodal-low” versus “bimodal-high” pollinators often led to an increase in the mismatch of reproductive-organ positions between patches, in reproductive isolation, and in adaptive divergence relative to controls (Fig. 3). These effects were strongest in biologically relevant conditions: weak stabilizing ecological selection [often observed empirically; 70,71,72], under all but the most imprecise pollen transfer dynamics, and at levels of geographic isolation from near-allopatry to near-sympatry. The effect size increased with increasing pollen transfer precision, suggesting that a better understanding of the precision of pollen transfer in natural systems is needed.

The traits that control reproductive-organ heights in the treatment realizations can be considered “magic” traits, that is, traits subject to divergent selection that also pleiotropically contribute to non-random mating [50], [51]. In heterostyly, these traits act as both mating cue and mating preference, an unusual situation [73]; the anther height in one morph is the cue, and the stigma at the same height in the reciprocal morph is the preference. Magic traits are theoretically important in speciation because they facilitate divergence among populations experiencing gene flow; specifically, a buildup of linkage disequilibrium is not necessary, because magic traits pleiotropically affect both fitness and reproductive isolation [50], [51], [74]. However, magic traits do not inevitably drive substantial divergence; rather, the effect size of a magic trait in ecological divergence and speciation might be expected to depend on both the strength of the divergent selection on it, and the strength of its effect on non-random mating [51], [75]. In the following paragraphs, we explore the above topics in relation to our model.

Divergent selection on reproductive-organ positions – the first half of their “magic trait” identity – is the result of sexual selection [62], generated by the particular pollinators present. Flowers with reproductive-organ positions that match the regions of greatest stickiness on the pollinators experience elevated male function (due to high pollen uptake) and/or elevated female function (due to high pollen receipt). The pollinators differ between populations, which thus generates divergent sexual selection on the reproductive-organ positions. Divergent selection due to divergent pollinator visitation preferences has been previously documented in putative magic traits [51]; however, our model is the first to explore the possibility of divergent selection due to mechanical differences among pollinators, without differences in pollinator behavior.

Non-random mating due to the reproductive-organ positions – the other half of their “magic trait” identity – is the result of precise pollen transfer: the greater the degree of precision in pollen transfer, the greater the tendency of flowers with reproductive-organ heights that (reciprocally) match to mate preferentially. The precision of pollen transfer in our model thus governs the “magicness”, or the effect size, of the magic traits [51], [75]. This result suggests that floral morphological traits that increase the precision of pollen transfer can increase the effect size of “magic” reproductive-organ–position traits, thus promoting speciation. We propose that such traits be called “magic modifiers”, since they modify the effect size of a magic trait, and therefore play a causal role in any resulting divergence and speciation. Pollinator traits, whether morphological or behavioral, that increase the precision of pollen transfer might also act as inter-genomic “magic modifiers”, with the potential for coevolutionary speciation dynamics.

As reproductive isolation evolves, gene flow decreases between patches, which allows further divergence in reproductive traits and thus the evolution of greater reproductive isolation. Ultimately, however, divergence in these magic traits is constrained by the pollinator stickiness functions, because divergence past the pollinator-determined optimal positions would result in a decrease in mating success due to decreased pollinator efficacy. The reproductive isolation here afforded by magic traits is thus limited, illustrating that speciation can be constrained by the same mechanisms that initially drive divergence, which is reminiscent of other cases of constraint due to conserved sexual selection [76]–[79]. This effect might represent a particular vulnerability of magic traits in driving speciation, since they pleiotropically control both local adaptation and reproductive isolation; if local adaptation demands a certain extent of divergence in the magic trait (but no more), then reproductive isolation might reach the corresponding level of non-random mating (but no more).

Although the definition of a “magic trait” simply stipulates that selection must be “divergent” between environments [51], these considerations show that the specific nature of that divergent selection will be essential to the outcome. If different environments exert opposing *directional* selection pressures on the magic trait, the potential exists for “runaway” divergence and speciation. If, however, different environments exert *stabilizing* selection on the magic trait favoring different optima, the outcome will depend on how divergent those optima are, and how much reproductive isolation the magic trait generates once the optima are attained. These observations underscore the centrality of the environment in the effect size of magic traits, which has been termed the “magic environment” perspective [75].

### Scenario 2: Asymmetrical gene flow and the evolution of dioecy

A scenario with “uniform” versus “high-biased” pollinators produced very different results from the preceding scenario; in this case, adaptive divergence was *less* in the treatment realizations than in the control realizations (Figs. 4G–I). This scenario might be quite biologically relevant, since the effect on (mal)adaptation within the two patches was strong even with imprecise pollen transfer, realistically weak ecological selection, and pollinator crossover probabilities ranging from near-allopatry to near-sympatry (Figs. 5A–F). The pollinators used here also appear to be quite biologically plausible (see Appendix S1, *Parameters*).

Adaptive divergence was reduced in the treatment realizations because asymmetries in pollen transport drive the pattern of mating among flowers (Fig. 5). Flowers in the first patch were served by the “uniform” pollinator, which transfers pollen equally well at both high and low positions. The “high-biased” pollinator in the second patch, however, picks up pollen at the high position (from thrums) much more effectively than at the low position (from pins), as found also in an empirical study [37]. In the second patch, therefore, thrums mostly remain unfertilized due to insufficient transported pin pollen. When pollinator crossover is infrequent (near allopatry), female reproductive function in patch 2 is markedly diminished (Figs. 5G–I). As the crossover probability rises, however, thrums in patch 2 increasingly become fertilized by pollen from patch 1's pins. Female function in patch 2 rises as a result (Figs. 5G–I), but the ensuing gene flow toward patch 2 causes strong ecological maladaptation (Figs. 5D–F).

Interestingly, this finding suggests that the population in patch 2 might experience an increase in fitness by eliminating the reproductive organs at the low height, since those organs serve chiefly as a vector for maladaptive gene flow. That is, given limited resources for reproductive effort, flowers in patch 2 that invest less in their low-position reproductive organs (but correspondingly more in their high-position reproductive organs) will have higher inclusive fitness, because a larger proportion of their offspring will be well-adapted. This idea suggests a novel mechanism for the evolution of dioecy from distyly by gender specialization [80], [81], related to the mechanism proposed by Beach and Bawa [82], but with additional selective pressure toward dioecy due to the maladaptive gene flow between populations. This modification seems likely to mitigate the objections of Muenchow and Grebus [83] that the mechanism of Beach and Bawa [82] works only under unrealistically stringent assumptions of a complete shift to a high-biased pollinator and a perfectly functioning genetic mechanism for loss of the low-position reproductive organs. Dioecy has independently evolved from distyly several times in the angiosperms, but the mechanism driving this transition remains unclear [82]–[85]; further research testing our hypothesized pathway could be informative.

### Broader implications

Results from our first scenario indicate that precise pollen transfer can cause partial reproductive isolation between populations that differ in their reciprocal reproductive-organ heights. This elevated reproductive isolation, even when small, often substantially increased adaptive divergence (Figs. 3E, 3H). Elevated reproductive isolation could increase the net diversification rate by promoting speciation, either directly by reducing gene flow [53], [86], or indirectly by promoting adaptive divergence that might generate further ecologically-driven reproductive isolation [87]–[89]. Additionally, the net diversification rate could increase due to the mitigation of extinction risk [35], either directly by promoting local adaptation that shields populations from extirpation [90]–[92], or indirectly via a “portfolio effect” resulting from a diversity of differently adapted populations [93]–[95]. Our results suggest that precise pollen transfer might therefore be responsible for the high diversity of some heterostylous clades.

Beyond heterostyly, our results have implications for the role of precision in pollen-transfer dynamics as a driver of reproductive isolation, adaptive divergence, and clade diversification in plants [96], as has been suggested in the cases of zygomorphic flowers [10], [19], [21] and flowers utilizing pollinia [97]. Even more generally, our results speak to the concepts of adaptive precision and accuracy [96], [98] in relation to traits influencing mate choice. A match between mating cues and preferences produces reproductive isolation in many systems, such as genital morphology in beetles [99], [100], color pattern preferences in cichlids [101], and song imprinting in birds [102], [103]. In all of these systems, the precision of cue–preference matching likely influences the strength of non-random mating, and thus the potential magic-trait effect size on speciation if the mating cue or preference is subjected to divergent selection; but the quantitative level of precision of cue–preference matching in such systems has rarely been considered.

Traits used as mating cues can diversify rapidly due to the interplay of assortative mating and ecological diversification, as observed in the systems mentioned above. This observation suggests a key prediction based on our results: clades for which precise pollen transfer promotes diversification should exhibit accelerated evolution of floral traits affecting anther and stigma position, due both to sexual selection exerted by different pollinators, and possibly also to reinforcement after secondary contact. In Bignonieae, Alcantara and Lohmann [104] found that patterns of interspecific variation among five floral traits governing reproductive-organ positions were congruent with evolutionary rates faster than drift-like Brownian motion, in contrast to the slow, conservative evolution found for the eleven other floral traits they studied. Bignonieae is a highly diverse clade with flowers possessing a relatively narrow corolla tube with the sexual organs concealed within, an architecture likely exerting control over the pollinator's positioning in order to promote precise pollen transfer [24]–[27], [58]. The results of Alcantara and Lohmann [104] thus suggest that precise pollen transfer has acted to promote diversification in Bignonieae. Congruently, de Vos et al. [105] found that distance between male and female sexual organ positions of heterostylous *Primula* flowers (with a floral architecture that similarly constrains flower-pollinator interaction) evolved at a 6-fold higher rate than those of largely or partially self-fertilizing monomorphic species, for which interactions with pollinators are less important.

Our second scenario differed from our first scenario only in the pollinators present. Nevertheless, it tells an entirely different story: that a pollinator that serves primarily high positions in the corolla tube can lead to strongly asymmetric gene flow between populations, causing substantial maladaptation [106] and producing strong selective pressure for innovations such as dioecy or a pollinator shift that would curtail the maladaptive gene flow. This very strong effect of pollinator morphology on evolutionary outcomes supports the idea that pollinator shifts might influence floral morphology and diversification [e.g., 107,108–110], even when pollinators differ only morphologically, not behaviorally. Furthermore, these observations might apply to any system in which female choice results in maladaptive gene flow from non-local males [76]–[79], [111], notably including human-disturbed systems [112].

The reproductive-organ–position traits that were magic and thus drove divergence in the first scenario failed to drive divergence in the second scenario, due only to an alteration in the pollinator milieu. This observation illustrates that magic traits do not exist in a vacuum; the effect size and indeed the very existence of a magic trait is influenced by factors external to the trait itself. That is, the existence and strength of divergent selection on a magic trait is a property of the environment, not of the trait, suggesting that there are “magic environments” [75] that cause “ordinary” traits that influence non-random mating to become magic. Similarly, our results suggest that the effect size of magic traits might be governed by other traits, “magic modifiers”, that influence the importance of the magic trait in non-random mating, such as floral traits that influence the precision of pollen transfer. A major question for speciation theory today is the origin of magic traits of large effect size – those that might drive speciation. In particular, do such traits arise by chance, or are they promoted in some manner by ecological and/or sexual selection? We suggest that magic environments, magic modifiers, and other factors external to the magic traits themselves might play an important role in answering this question.

Since our model is the first (to our knowledge) to simulate the movement of individual pollen grains between flowers and its effects on reproductive isolation and divergence, many promising directions exist for future work investigating the evolutionary ecology of pollen flow dynamics. Relaxing the assumption of one flower per plant would allow us to address questions involving geitonogamy, pollen discounting, and the evolutionary effects of different inflorescence types [113]-[116]. Modeling pollen carryover in longer visitation sequences [117], more complex mixed pollinator communities [9], [118], temporal variation in pollinators [119], and effects of pollinator behavior on pollen uptake [38] and positional transfer [120], could provide additional realism with important evolutionary effects. We could also extend our model to allow evolution in further floral traits, to explore phenomena such as the transition from distyly to dioecy (see *Scenario 2* above), the evolution of precise pollen transfer due to floral morphological traits such as corolla shape [24], and the quantitative evolution of reproductive strategies in response to pollination dynamics [121], [122]. Our results indicate that the mechanistic details of pollen flow, including the role of precise pollen transfer and the influence of pollinator morphology, can profoundly affect evolutionary outcomes, but these ideas have received little theoretical or empirical attention.

## Supporting Information

Figure S1 **The evolution of dimorphism in one patch, from the monomorphic initial state.** Colors indicate the value of the *S* trait; in this realization, red (*S* = 0) becomes thrum and blue (*S* = 1) becomes pin, but this polarity is emergent and random. Panels show a time series of model snapshots: 0 generations (A), 25 (B), 50 (C), 75 (D), 100 (E), 125 (F). Parameter values: *σ*_j_ = 0.1, *c* = 0.0, *ω* = 0.3, no pollinators (“control” run).
(TIFF)

Figure S2 **Effect of the precision of pollen transfer,** ***σ*** **_j_, on the final delivery height of pollen.** Panels show the three levels of pollen transfer precision used in model realizations (A: *σ*_j_ = 0.01, B: *σ*_j_ = 0.1, C: *σ*_j_ = 0.5). Dashed lines show three possible anther heights at which pollen is received by the pollinator. Solid curves show the relative frequency of pollen delivery at heights both within the corolla tube (yellow shading) and outside it. These results use the “uniform” pollinator; other pollinator functions will further affect the delivery height distribution. Very imprecise pollen transfer (panel C) shows that the center of the corolla tube is favored; this is due to the discarding of pollen grains that jitter beyond the corolla-tube limits during pickup (see Appendix S1, *Pollination phase*, step 8).
(TIFF)

Appendix S1 **A complete description of the individual-based model used.**
(PDF)
