# Supplementary figures and images for: A Tale of Two Morphs: Modeling Pollen Transfer, Magic Traits, and Reproductive Isolation in Parapatry
Source: PLoS One. 2014 Sep 11;9(9):e106512. doi: 10.1371/journal.pone.0106512 (PMC4161326; doi:10.1371/journal.pone.0106512)

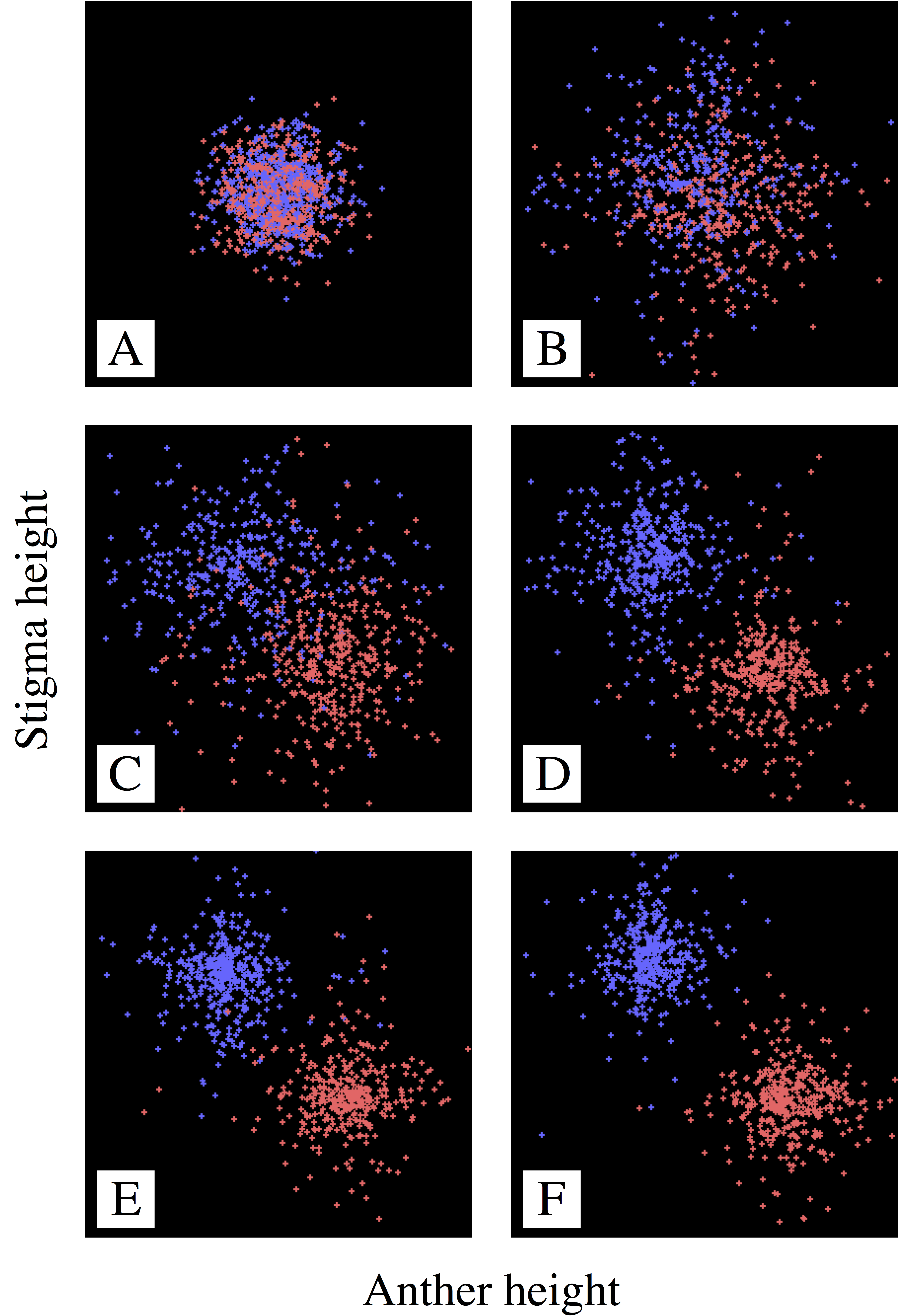

Supplement: Figure S1 — The evolution of dimorphism in one patch, from the monomorphic initial state. Colors indicate the value of the S trait; in this realization, red (S = 0) becomes thrum and blue (S = 1) becomes pin, but this polarity is emergent and random. Panels show a time series of model snapshots: 0 generations (A), 25 (B), 50 (C), 75 (D), 100 (E), 125 (F). Parameter values: σ j = 0.1, c = 0.0, ω = 0.3, no pollinators (“control” run). (TIFF) [file pone.0106512.s001.tiff]

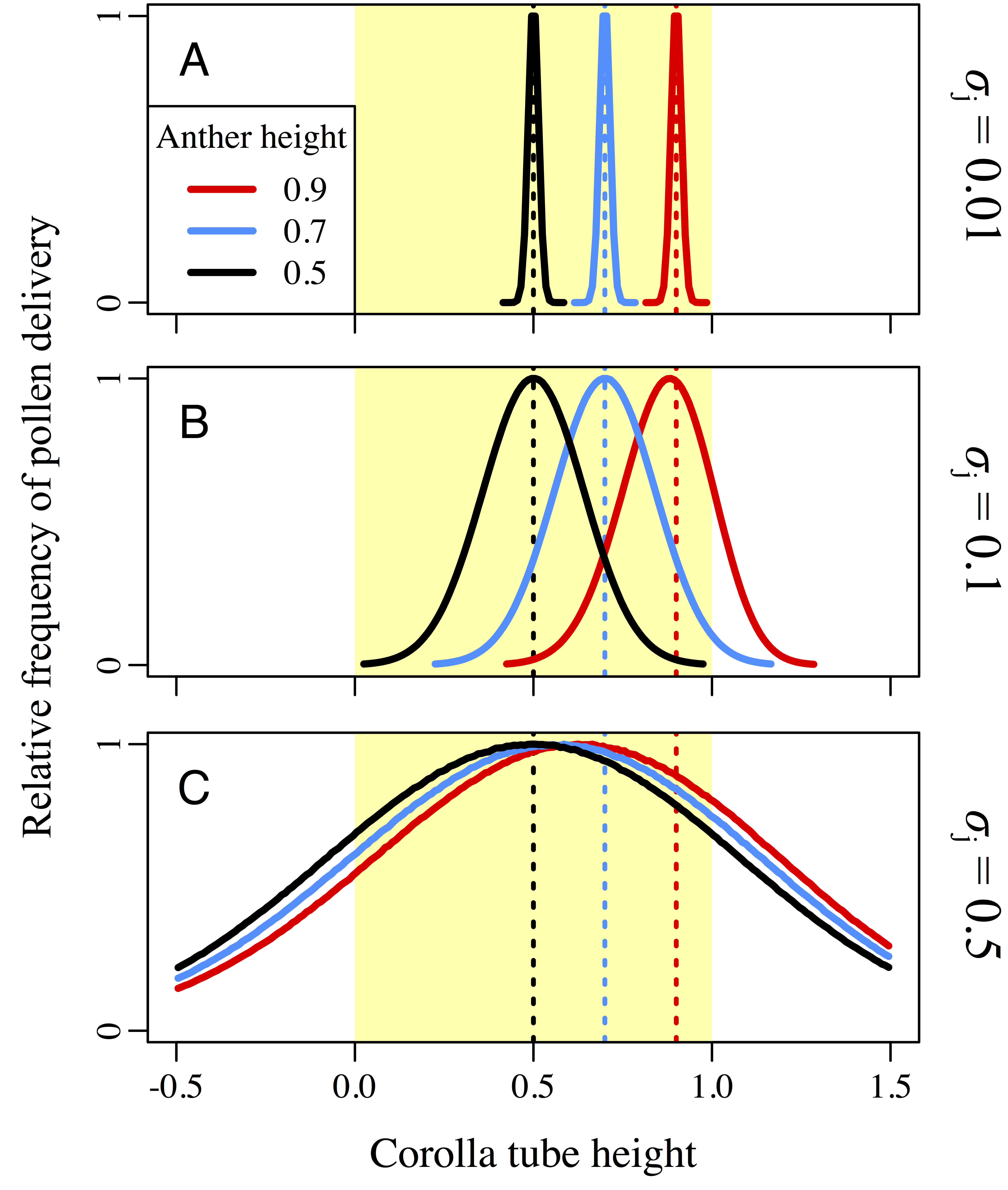

Supplement: Figure S2 — Effect of the precision of pollen transfer, σ j, on the final delivery height of pollen. Panels show the three levels of pollen transfer precision used in model realizations (A: σ j = 0.01, B: σ j = 0.1, C: σ j = 0.5). Dashed lines show three possible anther heights at which pollen is received by the pollinator. Solid curves show the relative frequency of pollen delivery at heights both within the corolla tube (yellow shading) and outside it. These results use the “uniform” pollinator; other pollinator functions will further affect the delivery height distribution. Very imprecise pollen transfer (panel C) shows that the center of the corolla tube is favored; this is due to the discarding of pollen grains that jitter beyond the corolla-tube limits during pickup (see Appendix S1, Pollination phase, step 8). (TIFF) [file pone.0106512.s002.tiff]
