# Supplementary material for: A Tale of Two Morphs: Modeling Pollen Transfer, Magic Traits, and Reproductive Isolation in Parapatry
Source: PLoS One. 2014 Sep 11;9(9):e106512. doi: 10.1371/journal.pone.0106512 (PMC4161326; doi:10.1371/journal.pone.0106512)
Supplement: Appendix S1 — A complete description of the individual-based model used. (PDF) [file pone.0106512.s003.pdf]

Supplemental: Model description

This model description follows conventions proposed by Grimm et al. [1] for the standardized presentation of individual-based models. All model parameters are listed in Table 1, while individual traits are given in Table 2. The model was implemented in the Objective-C language using Cocoa, an object-oriented framework (Apple Inc., http://www.apple.com, Mac OS X 10.7.4).

Purpose

The purpose of this model is to examine the evolutionary consequences of precise pollen transfer, using heterostylous flowers as its basis because this allows the effects of sexual interference to be separated from other phenomena. The model simulates the details of pollen transfer in sufficient detail to capture phenomena essential to heterostyly such as pollen wastage and style clogging, and it allows differences among pollinator species. Reproductive isolation due to spatial mismatch of reproductive organs between parapatric populations of heterostylous flowers can be an emergent consequence of the evolution of floral morphology in our model, and such reproductive isolation can, in turn, affect the extent of adaptation to the local environment of each population (Fig. 1).

Environment and state variables

Space in the model consists of two discrete patches of equal carrying capacity *K*. The patches differ along some unspecified ecological axis (e.g., wet/dry, warm/cold, etc.), as represented by a difference in the optimum ecological phenotype, θ_1 and θ_2, for individuals in the two environments. Each patch is inhabited by a single pollinator species, defined by a "pollinator function" that governs the probability that pollen will stick to the pollinator at a given height on its body. These functions are designated π_1 and π_2 for the two patches respectively; the pollinator may thus differ between the patches. The pollinators are stateless, do not evolve, choose randomly among flowers to visit, and are unaffected by events in the model.

Each patch is occupied by a population of individual heterostylous flowers (one flower per plant is assumed for simplicity). The heights of the anthers and stigma within the corolla tube are determined by two quantitative traits, x and y, constrained to lie within the boundaries of the corolla tube ($0 \leq x, y \leq 1$). The specific phenotypic effect of these traits depends upon an diallelic trait S not linked with x or y, inherited in a Mendelian fashion, that determines, as in real heterostylous flowers, whether a given flower is a "pin" (long-style "L-morph"), with a high stigma and low anthers, or a "thrum" (short-style "S-morph"), with a low stigma and high anthers (Fig. 1), and that produces the intra-morph self-incompatibility characteristic of heterostyly (see *Pollination phase*). In our model, the meaning of the S trait is not predetermined; whether values 0/1 represent pin/thrum or thrum/pin is emergent. The position of the stigma is represented by x if $S = 0$, or by y if $S = 1$; similarly, the position of the anthers is represented by x if $S = 1$, or by y if $S = 0$. This design produces morphologically reciprocal flowers with a change only in S; it also allows x and y to vary freely, rather than one having to be constrained to be greater than the other. Individuals also possess a quantitative "ecological trait", denoted z, that affects their local adaptation to their patch.

This genetic design is a reasonable approximation of what is known about the empirical genetics of heterostyly [2-5]. Heterostyly is governed by a locus (actually a supergene) called the S-locus that has, in effect, two alleles, s and S (actually two haplotypes, comprising several alleles at tightly linked loci); plants that are ss are pins, while plants that are Ss are thrums. Because only "legitimate" crosses – ss × Ss crosses – are typically allowed by the self-incompatibility system of heterostyly, empirical crosses typically produce equal proportions of ss and Ss offspring, and SS individuals do not normally exist in any substantial number; our representation of the S-locus with the two-valued trait S is thus reasonable (although extension of the model to allow illegitimate crosses, which do often occur in some heterostylous species, might provide further insights into the effects of unequal morph ratios on gene flow and

divergence). While the S-locus governs which reproductive organ appears at the high position and which appears at the low position in a given flower, the actual heights of the high and low positions seem likely to be governed by many loci of small effect size unlinked with the S-locus. Although the details of this are not known for any heterostylous species, Bissell and Diggle [6] did find that anther position and stigma position are genetically independent in species in *Nicotiana*; they argue that this independence provides useful evolvability to species in adapting to different pollinators. Other closely related heterostylous species are also known to exhibit differences in anther position, stigma position, and anther-stigma separation [e.g., 7], suggesting that the genetic independence found by Bissell and Diggle [6] may be general. We thus represent the heights of the reproductive organ positions as quantitative traits, x and y, with continuous values representing the additive effect of many alleles in an infinitesimal model [8,9]; while this architecture is not known to correspond to any particular heterostylous species, neither is it contradicted by any empirical findings of which we are aware, and it seems a simple and reasonable choice unlikely to bias our results in any important way. Environmental variance is not included in our model; in other words, the heritability of the traits is 1. Empirical evidence suggests that genetic variation and heritability for reproductive-organ positions is often quite high [10-15], so this seemed a reasonable choice for simplicity; evolution is likely more rapid in our model than it would be in real heterostylous systems as a result, but this does not affect our conclusions since we do not attempt to draw inferences regarding the absolute time required for adaptation. In any case, the stochasticity of the movement of pollen in our model (see *Pollination phase*) produces an effect very similar to the expected effect of environmental variance in reproductive-organ positions.

One consequence of our chosen genetic model is that the "polarity" of the S trait (whether $S = 0$ represents pin or thrum) is emergent: it is possible for the two populations to arrive at different polarities (almost always when the pollinator crossover probability was very low, since

gene flow pushes both patches toward a shared polarity). This resulted in almost complete reproductive isolation between patches, because morphs anatomically well-suited to exchange pollen (a pin in one patch and a thrum in the other) were blocked from cross-fertilizing by pollen incompatibility due to their possession of the same *S* trait value. This phenomenon of polarity differences among closely related heterostylous populations has not been observed in nature, likely due to shared ancestry [16]. For this reason, only realizations in which the two patches arrived at the same polarity were used; this constituted the large majority of realizations, since gene flow tended to cause the polarities in the two patches to synchronize. Removing realizations with opposing polarities between the patches amounts to enforcing a shared genetic architecture for heterostyly among the plants of the two patches, a reasonable assumption given that they represent diverging populations within the same species. Not removing realizations with opposing polarities, on the other hand, would produce incorrect results; our model would show very high degrees of reproductive isolation in those cases, but that isolation would be due to a mechanism that is not observed empirically because the genetic architecture of heterostyly is fixed by common ancestry. By removing these realizations, we focus attention on the empirically justified case, in which reproductive isolation between populations due to precise pollen transfer is the result of smaller, quantitative differences in reproductive organ heights, as shown in Fig. 1B.

The reproductive state of individuals is tracked with several non-genetic (i.e., non-heritable) state variables. Each individual has a number of pollen grains, p, which can be taken up and transported by pollinators, a number of ovules, o, that can be fertilized in each year, and a style clogging index, s, representing the empirical fact that the style of a flower becomes progressively clogged by pollen tubes that decrease the probability of fertilization. The details of these mechanics will be discussed below.

Processes and scheduling

Overlapping yearly generations of individuals are modeled as consisting of three phases executed consecutively in each year: germination, mortality, and pollination. In the germination phase, seedling individuals are generated from all fertilized ovules, and then seedlings die with a probability inversely proportional to their relative fitness in their local patch until the population size of each patch is less than or equal to the carrying capacity of the patch; conceptually, this represents the germination of seeds and the subsequent trait-dependent natural selection of seedlings during maturation. In the mortality phase, individuals die randomly with a fixed probability; this may be taken to represent either truly random mortality or natural selection on traits not modeled. In the pollination phase, the surviving individuals are visited by pollinators that transport pollen between them, resulting in the fertilization of ovules that will germinate at the beginning of the following year.

Interactions

In the germination phase, seedlings interact through competition in the sense that the probability of mortality for each seedling depends upon the number of other seedlings alive; in other words, natural selection during this phase is "soft selection" [17]. No interaction occurs during the mortality phase. In the pollination phase, each pollinator visit constitutes an interaction between two flowers, mediated by a pollinator, which can result in the depletion of the pollen reserve p of the donor flower, the increase of the style clogging index s of the recipient flower, and the fertilization of ovules o in the recipient flower. A pollinator visit can also result in the interaction of a flower with itself, because each flower visited by a pollinator can experience the transfer of self pollen from its anthers to its stigma as a result of the jostling of the flower by the pollinator. Details of these interactions will be provided in the description of each phase.

In this model, parapatric populations are connected due to "crossover" of pollinators between populations. In crossover events, a pollinator visits a flower in one patch, receiving

pollen, and then delivers that pollen to a flower in the other patch (and then returns to its native patch; see *Pollination phase* for further mechanistic details). These crossover events thus carry pollen between the populations, resulting in gene flow to the extent that that pollen successfully fertilizes flowers in the destination population; this gene flow is the only way in which the two populations interact. The crossover probability, c, ranges from complete allopatry ($c = 0.0$) to full sympatry ($c = 0.5$), although even in full sympatry the model separates the flowers into two discrete "patches" with distinct carrying capacity; this case corresponds to a heterogeneous local environment providing two ecologically distinct niches that are sufficiently spatially proximate as to produce no visitation bias in the pollinators. The crossover probability thus produces geographic isolation between populations in a similar manner to the behavioral isolation that would be produced by a pollinator preference for visitation of one type of plant over the other. Indeed, this model is nearly analogous to a fully sympatric model with two types of flowers differentiated by floral traits such as petal color that cause pollinators to exhibit visitation preferences [e.g., 18,19]. However, the distinct carrying capacity of the patches makes sense from a spatial perspective, but would be difficult to justify in a sympatric model of ethological isolation. For this reason, the crossover probability in this model controls geographic isolation, not ethological isolation, and the two framings are not equivalent.

Stochasticity

Stochasticity is present in many aspects of this model. The initial state of individuals is stochastic, such that the particular distribution of trait values slightly affects the speed with which dimorphism develops from the initial unimodal distribution, as well as the "polarity" of the S trait (whether $S = 0$ represents a pin or a thrum) once stable dimorphism is established. Demographic stochasticity is present due to finite population size, occasionally resulting in the extinction of one or both populations, and more generally affecting the evolutionary outcome of the model through drift. Stochasticity manifests in many aspects of pollination events (see

Pollination phase): the particular flowers visited, the number of pollen grains transported, whether each pollen grain sticks to the pollinator, the precision (or lack thereof) with which pollen grains are delivered at the same height at which they were picked up, whether each pollen grain is delivered to the destination flower's stigma, and whether each delivered pollen grain results in the fertilization of an ovule. Finally, stochasticity in the generation of offspring (due to mutation and to sexual reproduction) affects the phenotypes of offspring relative to their parents.

Observables

Several metrics were observed for each population in each generation of the realizations, including (1) the mean ecological trait value, (2) the mean values of the reproductive-organ–position traits, (3) the mean magnitude of herkogamy (mean absolute difference in height between anther and stigma), (4) the mean female reproductive fitness (proportion of ovules fertilized), (5) the mean male reproductive fitness (number of pollen grains that fertilized an ovule, normalized by dividing by the number of ovules per plant), (6) the proportion of pollen taken from anthers, (7) the extent of style clogging due to self pollen, illegitimate pollen (from the same morph, and thus blocked by the dimorphic incompatibility mechanism), and legitimate pollen (from the opposite, compatible morph), and (8) the magnitude of reproductive isolation at fertilization, calculated as the number of ovules fertilized by resident pollen divided by the total number of ovules fertilized, which combines the effects of geographic isolation with the effects of mechanical isolation due to sexual selection against non-local pollen. The mean value of the ecological trait in each patch, $\overline{z}_1$ and $\overline{z}_2$, at the end of each realization is particularly important because it allows us to evaluate the extent of local adaptation (or the lack thereof) exhibited by the plants in each patch as a consequence of the evolutionary dynamics experienced in each realization. All individuals were also observable graphically during model runs, including depictions of which flowers they were fertilized by and which flowers they fertilized, in order to allow both testing and exploration of the model [20].

Parameters

Parameter values governing the initial morphological distribution of the population (x_i, y_i, σ_{xi}^2, σ_{yi}^2) were chosen to generate a unimodal distribution of reproductive-organ positions normally distributed around the center of the corolla tube, with a variance similar to that observed for natural populations [7]. (Initializing the model with no initial variance, $\sigma_{xi}^2 = \sigma_{yi}^2 = 0$, appears to make no difference, however, as the same equilibrium variance is rapidly attained in any case.) Parameter z_i was chosen such that the initial population was equally maladapted ecologically (that is, with respect to the optimum) to both patches.

Parameter values governing the mutational variance (μ, α) were chosen to reproduce the same empirically observed variance in reproductive-organ position in subsequent generations. As is typical of individual-based models, a realistically low mutation rate would have resulted in the total loss of genetic variation at all loci, suggesting that processes other than mutation also act to maintain genetic variation in natural populations, but those processes are not well understood [21] and modeling them is in any case beyond the scope of the present research. The high mutation rate here, then, is equivalent to the fixed genetic variance assumed in many analytical models, and was not intended to be a realistic estimate of the mutation rate *per se*.

Parameter values governing the characteristics of the plants and patches (K, n_o, n_c, n_p, u_p, u_s, m, v, σ_p, σ_s, l_g, l_σ) were chosen with reference to personal observation of the "typical" values for heterostylous species in the genus *Primula*, but no attempt was made to precisely measure their values, or to make the model refer to any particular species. The model is not particularly sensitive to their values, although large changes to them can result in dynamics such as extreme pollen limitation that can have large effects.

The parameter values determining the difference in ecology between patches (θ_1, θ_2) were fixed, representing a standardized ecological difference of 1.0 between patches. The difference between θ_1 and θ_2 may be regarded as a scaling factor defining the meaning of both the ecological trait values, z, and the strength of natural selection, ω. Variation of ω thus explores the

full dimensionality of the parameter space here (given the initial condition of equal mean maladaptation to both patches).

Five parameters affecting the dynamics of natural selection due to ecology (ω) and sexual selection due to pollination (σ_j, c, π_1, π_2) were varied. The values used for these parameters are given in Table 1. Besides the values of σ_j listed, "control" realizations of the model were conducted that simulated pollen transfer with no precision in height whatsoever; see *Pollination phase* for more details.

The parameters π_1 and π_2 represent "pollinator functions" that give the probability that a pollen grain will stick to the pollinator at a given height h in the interval [0,1]. Four pollinator functions were used in the realizations presented, defined as

$$\pi_{\text{uniform}}(h) = 1\,,$$

$$\pi_{\text{high-biased}}(h) = \left\{ \begin{array}{cc} 0 & \text{for } h < 0.75 \\ 4(h-0.75) & \text{for } h \geq 0.75 \end{array} \right\},$$

$$\pi_{\text{bimodal-low}}(h) = \mathrm{N}_{0.03}(h-0.15) + \mathrm{N}_{0.03}(h-0.65)\text{, and}$$

$$\pi_{\text{bimodal-high}}(h) = \mathrm{N}_{0.03}(h-0.35) + \mathrm{N}_{0.03}(h-0.85)\,,$$

where $\mathrm{N}_\sigma(x) = e^{-x^2/2\sigma^2}$ denotes a Gaussian function with standard deviation σ. These functions are shown in Fig. 2. Since the "control" realizations of the model considered the height of transfer to be completely imprecise, no pollinator function was used in these runs; see *Pollination phase* for more details.

The biological relevance of the scenarios explored depends on the realism of the pollinator functions. Very few studies have examined how pollen sticks differentially to different pollinator body parts, or how pollinator behavior affects pollen pick-up and delivery positions. The best evidence for fine-scale differential stickiness is provided by Washitani et al. [22], in a detailed study of queen bumblebees pollinating *Primula sieboldii*. They document more than a 50-fold

difference in the number of pollen grains stuck to different proboscis regions, with strong spatial segregation of pin and thrum pollen; however, the contribution of differential stickiness remains unclear since pollen was presented only at naturally occurring anther heights. Other studies have generally focused on overall pollinator effectiveness, not fine-scale differential stickiness; nevertheless, these studies do indicate substantial variation in stickiness among body parts and some degree of precision in pollen transfer [23-30]. Studies have also documented evidence for different levels of stickiness at high versus low levels of the corolla tube [29,31], thus modulating pollen transfer differently between high and low reproductive organs, resulting in outcomes such as the loss of style polymorphism [32] and the evolution of dioecy [33], but the generality of these findings is unclear. In short, although evidence for differential stickiness on pollinators exists, very little is known about the details in particular systems, or about how this translates into a quantitative precision of pollen transfer. Since our results demonstrate that these details are important to evolutionary outcomes, further empirical work on pollen transfer dynamics is needed.

Initialization

Each patch was initially seeded with *K* adult individuals. Each individual's *x* value was drawn from a normal distribution with mean x_i and variance σ_{xi}^2, and the same was done for *y* values using y_i and σ_{yi}^2. The *z* values of all individuals were set to z_i with no variance; all variance in this trait stems from mutational variance introduced in later generations. The *S* value of each individual was chosen randomly with equal probabilities.

Initially, and at the beginning of the pollination phase of every subsequent year in the model, all individuals *i* were given n_p pollen grains ($p_i \leftarrow n_\mathrm{p}$), n_o unfertilized ovules ($o_i \leftarrow n_\mathrm{o}$), and an unclogged style ($s_i \leftarrow 0$).

Germination phase

At the beginning of the germination phase, some of the ovules of the flowers in each patch have been fertilized by pollen in the pollination phase of the preceding year (see *Pollination phase*). In the germination phase, those fertilized ovules become seedlings, and those seedlings compete for the opportunity to survive to adulthood.

As implemented, a fertilized ovule is essentially a contract between two parent plants to produce a seedling. The ovule knows the identity of the pollen donor plant *i*, as well as the identity of the pollen recipient *j* to which the ovule belongs. The seedling is generated directly given the genotypes of the parents, avoiding the need to explicitly represent gamete genotypes in the model. The offspring's trait values *x*, *y*, and *z* are drawn from normal distributions with means equal to the means of the parental trait values, $(x_i + x_j)/2$, $(y_i + y_j)/2$, and $(z_i + z_j)/2$, and standard deviations of half of the absolute difference in parental trait values, $|x_i - x_j|/2$, $|y_i - y_j|/2$, and $|z_i - z_j|/2$. This method of obtaining offspring trait values derives from Heinz et al. [34]; with it, the offspring's genotype depends only on the parental genotypes, in contrast to the standard quantitative genetics approach, in which the offspring's genotype depends upon the additive genetic variance of the whole population – the latter representing a violation of the "individual-based" paradigm. Like the standard quantitative genetics approach, our method correctly preserves the parental distribution of genetic values under random mating (R. Mazzucco, *pers. comm.*), and in a previous model it produced results similar to those obtained using other genetic architectures [35]. Because *x* and *y* are constrained to fall within the height range of the corolla tube, values outside of the interval [0,1] are redrawn until a permitted value is drawn; the value of *z* is unconstrained.

Each of the traits *x*, *y*, and *z* may then undergo mutation, each with a probability equal to the mutation rate μ. If a trait mutates, the value of the trait is offset by a draw from a normal distribution with mean 0 and standard deviation equal to the mutational effect size α (representing the effect of a mutation at a particular locus). Again, since traits *x* and *y* are

constrained, their mutational deviates are redrawn until the new value of the trait would lie within the interval [0,1].

Since one parent has $S = 0$ and one has $S = 1$, as enforced by the self-incompatibility mechanism of heterostyly mentioned previously, the value of the S locus of the offspring is randomly chosen as 0 or 1 with equal probabilities (modeling the empirical fact that a cross of Ss × ss produces, on average, 50% Ss and 50% ss).

All of the seedlings generated are added to the patch of their maternal parents. Competition then occurs, through soft selection based upon fitness derived from the ecological trait z, for the opportunity to mature to adulthood. If the total number of adult plants plus seedlings in a patch is less than or equal to the carrying capacity K, all seedlings survive to adulthood; otherwise, exactly enough seedlings will mature to fill the patch to carrying capacity. The probability of survival of each seedling i in patch j is proportional to its fitness W_i, as defined by a Gaussian function

$$W_i = e^{\frac{-(z_i-\theta_j)^2}{2\omega^2}},$$

where z_i is the ecological trait value of seedling i, θ_j is the optimum for patch j, and ω is the strength of natural selection. Seedlings that do not survive to adulthood die, and are removed from the patch.

Mortality phase

At the beginning of the mortality phase, each patch contains only adult plants. During this phase, each plant dies with probability m. Plants that die are removed from their patch.

Pollination phase

At the beginning of the pollination phase, the reproductive state of the survivors of the mortality phase is reset (see *Initialization*). Pollination events are then conducted consecutively

until the end of the pollination phase; the pollination season length v dictates the total number of pollination events conducted. Each pollination event consists of a set of steps executed sequentially:

(1) The patch of the donor flower, 0 or 1, is chosen with equal probabilities.

(2) A determination is made as to whether this pollination event is a "crossover", in which a pollinator visits one patch and then the other, using the crossover probability c. If it is not a crossover, the patch of the recipient flower is the same as the patch of the donor flower; if it is a crossover, the patch of the recipient flower is the other patch. (Note that pollinators that cross over do not then remain in their non-native patch; see step 16.)

(3) The donor flower i and the recipient flower j are chosen from their respective patches randomly, with equal probability given to every flower within a patch. If either patch is empty (due to the extinction of a population), or if the same flower was drawn as both donor and recipient, the pollination event terminates.

(4) Both flowers now undergo some degree of self pollination due to the jostling of the pollinator visit. This self pollination will not result in fertilization (since the self-incompatibility mechanism of heterostyly prevents that), but will result in pollen wastage and style clogging. The probability of a given pollen grain being jostled from anther to stigma of individual i (and, similarly, individual j) is determined by a scaled Gaussian function

$$P_{\text{jostle}} = u_s e^{\frac{-\left(h_{\text{pollen}} - h_{\text{stigma}}\right)^2}{2\sigma_s^2}},$$

where h_{pollen} is the height of the pollen grains (equal to the height of the anthers), h_{stigma} is the height of the stigma, σ_s is a scaling parameter determining how rapidly the probability of jostle transfer falls off with increasing difference between anther and

stigma heights, and u_s is the probability of jostle transfer for a flower with anther and stigma at the same height. Each available pollen grain in the anthers of flower i (of which there are p_i) is jostled to the stigma of the flower with probability P_{jostle}; the total number of pollen grains transferred is thus determined by a draw from a binomial distribution. Each pollen grain transferred is removed from the anthers ($p_i \leftarrow p_i - 1$), and results in the increment of the style clogging counter of the flower by 1 ($s_i \leftarrow s_i + 1$), representing one more pollen tube clogging the style.

(5) The pollinator π conducting the visit is chosen. With probability $1-c$ the pollinator is π_i, the pollinator of the donor flower's patch, while with probability c it is π_j, the pollinator of the recipient flower's patch. A given crossover pollination event can therefore be executed by either patch's native pollinator, but it is more likely to be executed by the pollinator that is native to the donor flower's patch, for the obvious reason that that pollinator is more likely to visit the donor flower's patch.

(6) The probability that a pollen grain will be removed from an anther by the pollinator is equal to the pollination uptake probability u_p [proportional removal, rather than numerical removal, sensu 36]. The number of pollen grains removed is thus determined by a draw from a binomial distribution with a number of trials equal to p_i, the number of pollen grains remaining in the anthers of individual i, and a per-trial probability of u_p. Each pollen grain taken up is removed from the anthers of the donor ($p_i \leftarrow p_i - 1$) and followed through the rest of its journey (steps 7–15, below). As implemented, each pollen grain is followed to completion before the next pollen grain is handled. The only consequence of this implementation detail is that when each pollen grain arrives at the recipient flower, the style clogging index and ovule state of that flower depends on the effects of the previous pollen grains delivered.

(7) For each pollen grain, it must now be determined whether it reaches the stigma of flower *j*. If this realization of the model is a "control" realization, delivery is guaranteed; skip to step 12. The essence of the control realizations, then, is that the height of the donor anthers and the recipient stigma are irrelevant; pollen transfer is completely imprecise. For the "treatment" realizations, delivery depends upon the height at which the pollen grain is received by the pollinator, the stickiness of the pollinator at that height, the height at which the pollen grain is delivered to the recipient, and the height of the recipient's stigma, as detailed in steps 8–11.

(8) Each pollen grain removed from the donor is received by the pollinator at the same height on the pollinator's body, deviated by a draw from a normal distribution with mean 0 and standard deviation σ_{j} representing stochasticity in the transfer. If the resulting height is outside of the interval [0,1], the pollen grain is presumed to have been lost, and its transfer is terminated.

(9) Each pollen grain received by the pollinator at height h has a probability of sticking to the pollinator, defined by the pollinator stickiness function $\pi(h)$. If the pollen does not stick, it is presumed lost.

(10) Each pollen grain stuck to the pollinator is delivered to the recipient flower at a height equal to the height at which it stuck to the pollinator, deviated by a draw from a normal distribution with mean 0 and standard deviation σ_{j} representing stochasticity in the transfer. The delivery height is allowed to be outside the [0,1] interval.

(11) For each pollen grain delivered, the probability that it is received by the recipient flower's stigma is determined by a Gaussian function

$$P_{\mathrm{receipt}} = e^{\frac{-\left(h_{\mathrm{pollen}} - h_{\mathrm{stigma}}\right)^2}{2\sigma_p^{\,2}}},$$

where h_{pollen} is the height at which the pollen grain is delivered, h_{stigma} is the height of the stigma of the recipient flower, and σ_{p} is a scaling parameter determining how rapidly the probability of receipt of a pollen grain falls off with increasing difference between delivery height and stigma height.

(12) Each delivered pollen grain causes the increment of the recipient flower's style clogging counter by one ($s_j \leftarrow s_j + 1$), regardless of the compatibility of the pollen grain; it is assumed that all pollen tubes, whether compatible or not, produce the same degree of clogging of the style.

(13) If the delivered pollen grain is incompatible (conceptually, thrum pollen landing on a thrum flower or pin pollen landing on a pin flower; more precisely, if $S_i = S_j$, but see *Environment and state variables* regarding the polarity of the S trait), the pollen grain is now discarded. The only side effects of incompatible pollination events are thus the removal of pollen from the donor and style clogging in the recipient.

(14) Each compatible pollen grain delivered to the recipient stigma now has a probability of fertilizing an ovule (assuming one is available, $o_j > 0$) that decreases with the proportion of style clogging experienced by the recipient flower: $P_{\text{fertilization}} = 1 - s_j / n_s$, where s_j is the style clogging counter of the recipient flower and n_{s} is the style clogging limit at which fertilization is completely blocked. (If $s_j > n_{\text{s}}$, $P_{\text{fertilization}}$ is taken to be zero.)

(15) Each ovule fertilized is removed from the pool of unfertilized ovules in the recipient ($o_j \leftarrow o_j - 1$). Each fertilized ovule remembers the donor flower that fertilized it, and will generate a seedling in the germination phase of the following year.

(16) At the end of each pollination event, a pollinator that has crossed over is assumed to return to its native patch (or to die, which amounts to the same thing since the supply of pollinators in the model is unlimited). This assumption is necessary, since otherwise

the pollinators would soon equilibrate at equal frequency in both patches, undermining the very idea of a different pollinator native to each patch.

The preceding description of a pollination event uses Gaussian functions for the probability that a pollen grain at height h_{pollen} will be received by a stigma at h_{stigma}, during both self-pollination (step 4) and pollen delivery (step 11). However, since gravity would tend to pull pollen grains downward, the probability of pollen receipt might more closely resemble a lognormal function with its maximum where $h_{\mathrm{pollen}} = h_{\mathrm{stigma}}$. A pollen grain that arrives at or below a threshold l_{g} below the stigma has a probability of zero of being received because pollen doesn't fall upward (the lognormal function is undefined here, but is taken to be zero). Above the maximum value of the function at $h_{\mathrm{pollen}} = h_{\mathrm{stigma}}$, the probability of receipt falls off asymptotically toward zero at a rate defined by the scale parameter of the lognormal, l_{o}, reflecting the possibility that pollen can fall downward from any height above the stigma and have some nonzero probability of being received by it.

A version of the model incorporating this lognormal-based pollen delivery was constructed. Specifically, in this version of the model the formula in step 4 is replaced by

$$P_{\mathrm{jostle}} = \left\{ \begin{array}{cc} u_s \dfrac{\mathrm{L}(l_{\mathrm{g}} + h_{\mathrm{pollen}} - h_{\mathrm{stigma}}, l^2{}_{\sigma} + \ln\left(l_{\mathrm{g}}\right), l_{\sigma})}{\mathrm{L}\left(l_{\mathrm{g}}, l^2{}_{\sigma} + \ln\left(l_{\mathrm{g}}\right), l_{\sigma}\right)} & \text{for } h_{\mathrm{pollen}} - h_{\mathrm{stigma}} > -l_{\mathrm{g}} \\ 0 & \text{otherwise} \end{array} \right\}$$

and the formula in step 11 is replaced by

$$P_{\mathrm{receipt}} = \left\{ \begin{array}{cc} \dfrac{\mathrm{L}(l_{\mathrm{g}} + h_{\mathrm{pollen}} - h_{\mathrm{stigma}}, l^2{}_{\sigma} + \ln\left(l_{\mathrm{g}}\right), l_{\sigma})}{\mathrm{L}\left(l_{\mathrm{g}}, l^2{}_{\sigma} + \ln\left(l_{\mathrm{g}}\right), l_{\sigma}\right)} & \text{for } h_{\mathrm{pollen}} - h_{\mathrm{stigma}} > -l_{\mathrm{g}} \\ 0 & \text{otherwise} \end{array} \right\},$$

where $\mathrm{L}(x, \mu, \sigma)$ is the standard lognormal function

$$L(x,\mu,\sigma)=\frac{1}{x\sigma\sqrt{2\pi}}e^{\frac{-(\ln(x)-\mu)^2}{2\sigma^2}}.$$

The quotient form of the formulas for P_{jostle} and P_{receipt} serves to normalize the height of the functions to 1 when $h_{\text{pollen}} = h_{\text{stigma}}$. The value $l^2{}_\sigma + \ln(l_g)$ for the μ parameter of the lognormals serves to locate the peak of the functions at $x = l_g$.

Results from the lognormal version of the model were generally qualitatively similar to results from the Gaussian version of the model. In some ways the lognormal model did appear to better match empirical data; in particular, thrums received more self pollen than pins in this version of the model, increasing their male function and decreasing their female function relative to pins, a phenomenon which has often been observed in nature [37-39]. These dynamics did not substantially affect the results of the model presented here, however, and so all results presented are taken from the Gaussian version of the model, since it is conceptually simpler. In general, the dynamics of pollination are remarkably complex [e.g., 40,41,42], and attempting to introduce all of this complexity into a model would be premature; we have strived for a balance that includes only that complexity necessary to pursue the questions at hand.

References

1. Grimm V, Berger U, Bastiansen F, Eliassen S, Ginot V, et al. (2006) A standard protocol for describing individual-based and agent-based models. Ecological Modelling 198: 115–126.

2. Barrett SCH, Shore JS (2008) New insights on heterostyly: Comparative biology, ecology and genetics. In: Franklin-Tong VE, editor. Self-Incompatibility in Flowering Plants – Evolution, Diversity, and Mechanisms. Berlin: Springer-Verlag. pp. 3–32.

3. Labonne JDJ, Vaisman A, Shore JS (2008) Construction of a first genetic map of distylous *Turnera* and a fine-scale map of the *S*-locus region. Genome 51: 471–478.

4. Labonne JDJ, Tamari F, Shore JS (2010) Characterization of X-ray-generated floral mutants carrying deletions at the *S*-locus of distylous *Turnera subulata*. Heredity 105: 235–243.

5. Gilmartin PM, Li J (2010) Homing in on heterostyly. Heredity 105: 161–162.

6. Bissell EK, Diggle PK (2010) Modular genetic architecture of floral morphology in *Nicotiana*: Quantitative genetic and comparative phenotypic approaches to floral integration. Journal of Evolutionary Biology 23: 1744–1758.

7. Keller B, de Vos JM, Conti E (2012) Decrease of sexual organ reciprocity between heterostylous primrose species, with possible functional and evolutionary implications. Annals of Botany 110: 1233–1244.

8. Fisher RA (1918) The correlation between relatives on the supposition of Mendelian inheritance. Transactions of the Royal Society of Edinburgh 52: 399–433.

9. Falconer DS (1989) Introduction to Quantitative Genetics. New York, NY: John Wiley & Sons, Inc. 438 p.

10. Shore JS, Barrett SCH (1990) Quantitative genetics of floral characters in homostylous *Turnera ulmifolia* var. *angustifolia* Willd. (Turneraceae). Heredity 64: 105–112.

11. Carr DE, Fenster CB (1994) Levels of genetic variation and covariation for *Mimulus* (Scrophulariaceae) floral traits. Heredity 72: 606–618.

12. Kulbaba MW, Worley AC (2008) Floral design in *Polemonium brandegei* (Polemoniaceae): Genetic and phenotypic variation under hawkmoth and hummingbird pollination. International Journal of Plant Sciences 169: 509–522.

13. Herlihy CR, Eckert CG (2007) Evolutionary analysis of a key floral trait in *Aquilegia canadensis* (Ranunculaceae): Genetic variation in herkogamy and its effect on the mating system. Evolution 61: 1661–1674.

14. Bartkowska MP, Johnston MO (2009) Quantitative genetic variation in populations of *Amsinckia spectabilis* that differ in rate of self-fertilization. Evolution 63: 1103–1117.

15. Ashman T-L, Majetic CJ (2006) Genetic constraints on floral evolution: A review and evaluation of patterns. Heredity 96: 343–352.

16. Mast AR, Conti E (2006) The primrose path to heterostyly. New Phytologist 171: 439–442.

17. Wallace B (1975) Hard and soft selection revisited. Evolution 29: 465–473.

18. Campbell DR, Waser NM, Pederson GT (2002) Predicting patterns of mating and potential hybridization from pollinator behavior. American Naturalist 159: 438–450.

19. Gegear RJ, Burns JG (2007) The birds, the bees, and the virtual flowers: Can pollinator behavior drive ecological speciation in flowering plants? American Naturalist 170: 551–566.

20. Grimm V (2002) Visual debugging: A way of analyzing, understanding and communicating bottom-up simulation models in ecology. Natural Resource Modeling 15: 23–38.

21. Barton NH, Keightley PD (2002) Understanding quantitative genetic variation. Nature Reviews Genetics 3: 11–21.

22. Washitani I, Kato M, Nishihiro J, Suzuki K (1995) Importance of queen bumble bees as pollinators facilitating inter-morph crossing in *Primula sieboldii*. Plant Species Biology 9: 169–176.

23. Olesen JM (1979) Floral morphology and pollen flow in the heterostylous species *Pulmonaria obscura* Dumort (Boraginaceae). New Phytologist 82: 757–767.

24. Lewis D (1982) Incompatibility, stamen movement and pollen economy in a heterostyled tropical forest tree, *Cratoxylum formosum* (Guttiferae). Proceedings of the Royal Society Biological Sciences Series B 214: 273–283.

25. Lau P, Bosque C (2003) Pollen flow in the distylous *Palicourea fendleri* (Rubiaceae): An experimental test of the Disassortative Pollen Flow Hypothesis. Oecologia 135: 593–600.

26. Massinga PH, Johnson SD, Harder LD (2005) Heteromorphic incompatibility and efficiency of pollination in two distylous *Pentanisia* species (Rubiaceae). Annals of Botany 95: 389–399.

27. Pérez-Barrales R, Vargas P, Arroyo J (2006) New evidence for the Darwinian hypothesis of heterostyly: Breeding systems and pollinators in *Narcissus* sect. Apodanthi. New Phytologist 171: 553–567.

28. Brys R, Jacquemyn H, Hermy M, Beeckman T (2008) Pollen deposition rates and the functioning of distyly in the perennial *Pulmonaria officinalis* (Boraginaceae). Plant Systematics and Evolution 273: 1–12.

29. Keller B, Thomson JD, Conti E (2014) Heterostyly promotes disassortative pollination and reduces sexual interference in Darwin's primroses: Evidence from experimental studies. Functional Ecology, DOI: 10.1111/1365-2435.12274.

30. Stone JL, Thomson JD (1994) The evolution of distyly: Pollen transfer in artificial flowers. Evolution 48: 1595–1606.

31. Simon-Porcar VI, Santos-Gally R, Arroyo J (2014) Long-tongued insects promote disassortative pollen transfer in style-dimorphic Narcissus papyraceus (Amaryllidaceae). Journal of Ecology 102: 116–125.

32. Pérez-Barrales R, Arroyo J (2010) Pollinator shifts and the loss of style polymorphism in *Narcissus papyraceus* (Amaryllidaceae). Journal of Evolutionary Biology 23: 1117–1128.

33. Beach JH, Bawa KS (1980) Role of pollinators in the evolution of dioecy from distyly. Evolution 34: 1138–1142.

34. Heinz SK, Mazzucco R, Dieckmann U (2009) Speciation and the evolution of dispersal along environmental gradients. Evolutionary Ecology 23: 53–70.

35. Haller BC, Hendry AP (2013) Solving the paradox of stasis: Squashed stabilizing selection and the limits of detection. Evolution 68: 483–500.

36. Harder LD, Thomson JD (1989) Evolutionary options for maximizing pollen dispersal of animal-pollinated plants. American Naturalist 133: 323–344.

37. Nicholls MS (1986) Population composition, gender specialization, and the adaptive significance of distyly in *Linum perenne* (Linaceae). New Phytologist 102: 209–217.

38. Casper BB, Charnov EL (1982) Sex allocation in heterostylous plants. Journal of Theoretical Biology 96: 143–149.

39. Lloyd DG (1979) Evolution towards dioecy in heterostylous populations. Plant Systematics and Evolution 131: 71–80.

40. Kudo G (2003) Anther arrangement influences pollen deposition and removal in hermaphrodite flowers. Functional Ecology 17: 349–355.

41. Armbruster WS, Pérez-Barrales R, Arroyo J, Edwards ME, Vargas P (2006) Three-dimensional reciprocity of floral morphs in wild flax (*Linum suffruticosum*): A new twist on heterostyly. New Phytologist 171: 581–590.

42. Wolfe LM, Barrett SCH (1989) Patterns of pollen removal and deposition in tristylous *Pontederia cordata* L. (Pontederiaceae). Biological Journal of the Linnean Society 36: 317–329.
